# Machine learning-based radiomics analysis of preoperative functional liver reserve with MRI and CT image

**DOI:** 10.1186/s12880-023-01050-1

**Published:** 2023-07-17

**Authors:** Ling Zhu, Feifei Wang, Xue Chen, Qian Dong, Nan Xia, Jingjing Chen, Zheng Li, Chengzhan Zhu

**Affiliations:** 1 Shandong Key Laboratory of Digital Medicine and Computer Assisted Surgery, The Affiliated Hospital of Qingdao University, Qingdao, China; 2 Institute for Digital Medicine and Computer-assisted Surgery in Qingdao University, Qingdao University, Qingdao, China; 3 Department of Pediatric Surgery, The Affiliated Hospital of Qingdao University, Qingdao, China; 4 Department of Radiology, The Affiliated Hospital of Qingdao University, Qingdao, China; 5 Qingdao Hisense Medical Equipment Co., Ltd, Qingdao, China; 6 Department of Hepatobiliary and Pancreatic Surgery, The Affiliated Hospital of Qingdao University, Qingdao, China

**Keywords:** Radiomics, Functional liver reserve, Machine learning, Gd-EOB-DTPA-enhanced hepatic MRI, Contrast-enhanced CT

## Abstract

**Objective:**

The indocyanine green retention rate at 15 min (ICG-R15) is a useful tool to evaluate the functional liver reserve before hepatectomy for liver cancer. Taking ICG-R15 as criteria, we investigated the ability of a machine learning (ML)-based radiomics model produced by Gd-EOB-DTPA-enhanced hepatic magnetic resonance imaging (MRI) or contrast-enhanced computed tomography (CT) image in evaluating functional liver reserve of hepatocellular carcinoma (HCC) patients.

**Methods:**

A total of 190 HCC patients with CT, among whom 112 also with MR, were retrospectively enrolled and randomly classified into a training dataset (CT: n = 133, MR: n = 78) and a test dataset (CT: n = 57, MR: n = 34). Then, radiomics features from Gd-EOB-DTPA MRI and CT images were extracted. The features associated with the ICG-R15 classification were selected. Five ML classifiers were used for the ML-model investigation. The accuracy (ACC) and the area under curve (AUC) of receiver operating characteristic (ROC) with 95% confidence intervals (CI) were utilized for ML-model performance evaluation.

**Results:**

A total of 107 different radiomics features were extracted from MRI and CT, respectively. The features related to ICG-R15 which was classified into 10%, 20% and 30% were selected. In MRI groups, classifier XGBoost performed best with its AUC = 0.917 and ACC = 0.882 when the threshold was set as ICG-R15 = 10%. When ICG-R15 = 20%, classifier Random Forest performed best with AUC = 0.979 and ACC = 0.882. When ICG-R15 = 30%, classifier XGBoost performed best with AUC = 0.961 and ACC = 0.941. For CT groups, the classifier XGBoost performed best when ICG-R15 = 10% with AUC = 0.822 and ACC = 0.842. When ICG-R15 = 20%, classifier SVM performed best with AUC = 0.860 and ACC = 0.842. When ICG-R15 = 30%, classifier XGBoost performed best with AUC = 0.938 and ACC = 0.965.

**Conclusions:**

Both the MRI- and CT-based machine learning models are proved to be valuable noninvasive methods for functional liver reserve evaluation.

**Supplementary Information:**

The online version contains supplementary material available at 10.1186/s12880-023-01050-1.

## Introduction

Primary liver cancer is the sixth most common cancer and is demonstrated to be the third contributing factor for global cancer death, showing about 906,000 new cases and 830,000 deaths in 2020 [1]. Among primary liver cancer cases, hepatocellular carcinoma (HCC) accounts for the most with about 75-85% [1]. Evidence has shown that Asia and Africa get the highest incidence of HCC in the world [2]. For HCC therapy, partial hepatectomy (PH) is still the optimal choice although most patients have reached an advanced stage because of insidious symptoms [3, 4]. However, it should be noticed that the post-hepatectomy liver failure (PHLF) is one of the important complications, and PHLF is the major cause of postoperative mortality. Normally the incidence of PHLF is 0.7-9.1% and can reach 58.22% when the major hepatectomy is performed [5, 6]. Thus the presurgical evaluation of functional liver reserve seems critical and necessary as the accurate evaluation can help reduce the risk of hepatectomy and avoid PHLF.

In clinical work, liver volumetry and scoring systems based on blood tests, such as ALB, AST, TBIL, and indocyanine green retention at 15 min retention rate (ICG-R15) are classic indexes used for the evaluation of functional liver reserve. Liver volumetry can be obtained by 3D reconstruction technology [7]. The scoring systems contain the MELD score, Child-Turcotte-Pugh (CTP) score, and Albumin-bilirubin (ALBI) grade. By comparing the above indexes, the ICG-R15 has its own advantages. Firstly, it can help doctors detect functional liver reserve abnormality earlier and more accurately. Secondly, it has also been proven to have a positive correlation to liver failure and morbidity after hepatectomy [8]. The ICG-R15 values of the patient in different intervals (threshold: 10%, 20%, 30%) can affect and guide the selection of surgical treatment methods [9]. Thus, the ICG clearance test was considered to be the optimal evaluation of preoperative function liver reserve [10].

Radiomics, which is one emerging methodology in medicine, can depict quantitative computerized algorithm-based features from traditional image materials, like CT or MRI images [11–13]. The medical images were proven related to clinical manifestations and the relation can be identified via machine learning (ML) approaches [14, 15]. In the past studies, the radiomics has been applied to investigating liver diseases, such as diagnosis, staging, liver tumor biological behaviors, and prognosis of primary liver cancer [16, 17]. Radiomics is promising to assist doctors in evaluating hepatic functional reserve, especially in conditions of the lack of ICG equipment in the hospital or other reasons that cause the failure of the ICG clearance test. However, the value of radiomics in evaluating functional liver reserve has been scarcely examined.

Under this direction, our work will evaluate whether the radiomics models derived from Gd-EOB-DTPA-enhanced hepatic MRI or contrast-enhanced CT can assess functional liver reserve and will further compare their performance with ICG-R15 in HCC patients.

## Methods

### Flowchart

**Fig. 1:**
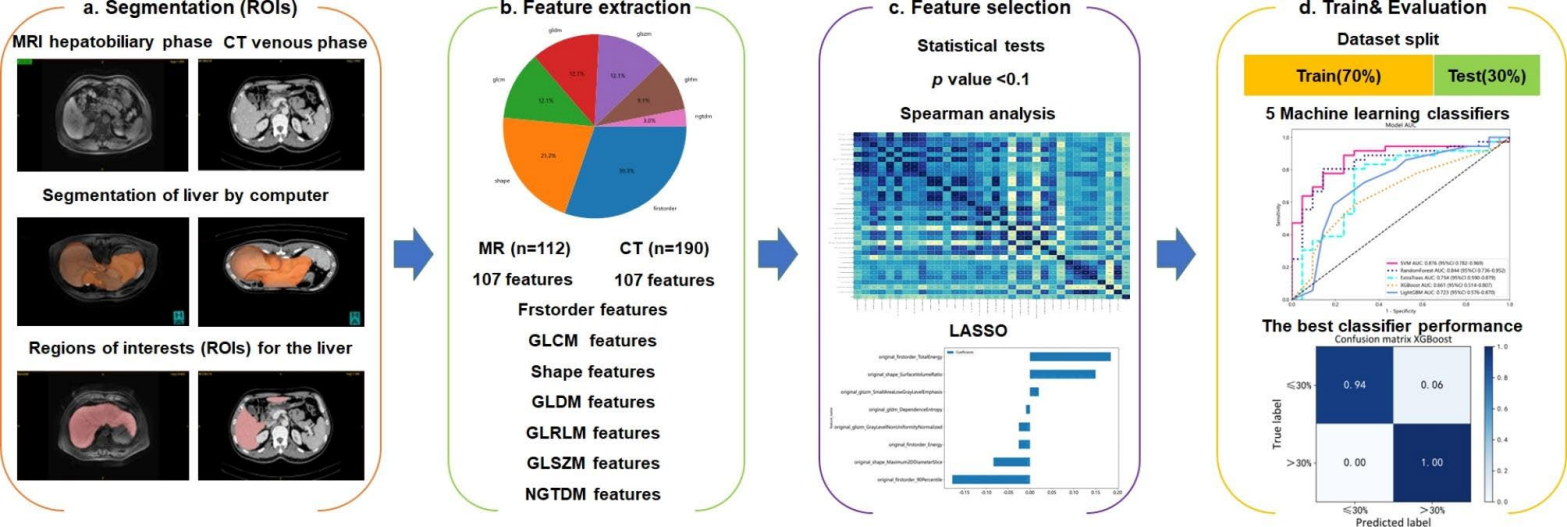
Depiction of the whole procedure for developing and validation of the machine learning models

According to the flowchart of this work (Fig. 1), the Gd-EOB-DTPA-enhanced hepatic MRI (MRI) data from 112 patients and contrast-enhanced CT (CT) data from 190 patients were retrospectively collected. The hepatobiliary phase at 15 min of MRI and the portal venous phase of CT were selected. Firstly, the ROI liver region was segmented, and the features were extracted from the MRI and CT images. Next, the features were further screened through the P-value and correlation coefficient. For radiomic model development, the dataset was randomly divided into a training dataset and a test dataset. The training dataset aimed to train the model. Before training, the features associated with the label of ICG-R15 were screened by using the least absolute shrinkage and selection operator (LASSO) algorithm. Multiple ML algorithms were used for training on the training dataset. The training models based on the multiple ML algorithms will be further examined to classify the functional liver reserve on the test dataset. At last, the best model was selected to evaluate functional liver reserve classification.

### Patients

By reviewing the patients who were diagnosed with HCC in our hospital from May 2017 to April 2022, the inclusion criteria were set as follows: (1) all patients were diagnosed as HCC; (2) Gd-EOB-DTPA-enhanced hepatic MRI or contrast-enhanced CT in all phases was completed within one week before treatment or surgery; (3) ICG clearance test was completed within one week before treatment or surgery; (4) patients without jaundice during ICG clearance test [18]; (5) all patients had no history of previous liver surgery or radiofrequency ablation(RFA). A total of 190 patients were included in this study. All the 190 patients have CT data while only 112 cases of them have both MRI data. Details are listed in Table 1. The Ethics Committee of the Affiliated Hospital of Qingdao University approved this study with the ethical approval number QYFY-WZLL-27,465.

**Table 1:** Demographics and preoperative data of patients

Characteristics	Categories	Value		Number	
		MRI	CT	MRI	CT
dataset	Training dataset (70%)			78	133
	Test dataset (30%)			34	57
Age (mean ± SD)		58.62 ± 8.62 y	59.42 ± 8.93 y		
Gender	Female			25	44
	Male			87	146
HBV infection	Yes			104	168
	No			8	22
Liver cirrhosis	Yes			75	122
	No			37	68
BMI (mean ± SE)		25.02 ± 0.39 kg/m2	24.75 ± 0.27 kg/m2		
TBIL (mean ± SE)		21.32 ± 1.60 µmol/L	22.37 ± 1.10 µmol/L		
ALB (mean ± SE)		36.26 ± 0.66 g/L	36.99 ± 0.43 g/L		
ALT (mean ± SE)		48.38 ± 10.21 IU/L	49.06 ± 5.96 IU/L		
PT (mean ± SE)		11.34 ± 0.14s	11.45 ± 0.11s		
AST (mean ± SE)		52.31 ± 6.81 IU/L	56.08 ± 5.24 IU/L		
GGT (mean ± SE)		99.44 ± 16.15 IU/L	108.12 ± 12.23 IU/L		
ICG-R15	ICG-R15 ≤ 10% vs. ICG-R15>10%			62vs128	45vs67
	ICG-R15 ≤ 20% vs. ICG-R15>20%			126vs64	78vs34
	ICG-R15 ≤ 30% vs. ICG-R15>30%			160vs30	93vs19

ALT: alanine transaminase, BMI: Body Mass Index, HBV: Hepatitis B Virus; TBIL: total Bilirubin, ALB: albumin, PT: prothrombin time, AST: aspartate aminotransferase, GGT: gamma-glutamyltransferase, ICG-R15: indocyanine green retention rate at 15 min, y: years

### ICG Clearance Test

After 6 h of fasting, the patient was in a supine position and injected 0.5 mg/kg ICG (Dandong Yichuang Pharmaceutical Co., Ltd., Liaoning, China) intravenously into a peripheral vein within 10 s. The ICG retention rate was measured with ICG pulse spectrophotometry (DDG 3300 K, Japan) after 15 min of injection. The ICG-R15 value was expressed as the percentage of ICG retention in serum 15 min after injection.

### MRI and CT acquisition

The Gd-EOB-DTPA-enhanced hepatic MRI examination and the contrast-enhanced CT examination were conducted by a Siemens Skyra 3.0 T MRI scanner and a Siemens SOMATOM Definition Flash scanner, respectively. Scans were performed from the top of the liver to the pelvis. The MRI scanning parameters were selected as below: the repetitiontime was 3.9 ms, the echotime was 1.4 ms, the matrix was 320 × 256, the field of view was 400 mm × 400 mm and the slice thickness was 3 mm. The Gd-EOB-DTPA (Primovist, Bayer Schering Pharma AG, Berlin, Germany) was used as a contrast agent for enhanced MRI scanning, and the contrast agent (with flow rate of 2.0 ml/s and dose of 0.1ml/kg) was injected through elbow vein using a high-pressure syringe. Afterwards, 20 ml of saline is flushed. The arterial phase, portal venous phase, transitional phase and hepatobiliary phase were acquired at 30 s, 70 s, 180 s and 15 min, respectively. The CT scanning parameters were selected as below: the voltage was 120 kV, the current was 100–400 mA, the layer thickness was 5 mm, the layer spacing was 5 mm, the slice thickness was 1 mm and the matrix was 512 × 512. The iohexol (Yangtze River Pharmaceutical Group, Jiangsu, China) was used as a contrast agent for enhanced CT scanning. The contrast agent (with flow rate of 3.0 ml/s and dose of 1.5 ml/kg) was injected through the peripheral vein using a double-barreled high-pressure syringe. The arterial phase, portal venous phase and equilibrium phase were acquired at 30 s, 60 and 180 s, respectively.

### Image segmentation

The computer-assisted surgery system (CAS) (CAS-Lv, Qingdao Hisense Medical Equipment Co., Ltd.) was used to segment the liver contour automatically from the hepatobiliary phase after 15 min of MRI and venous phase of the CT, to obtain the regions of interest (ROI) of the liver. For liver segmentation of MRI and CT, the Dice coefficient was more than 0.95 (this Dice coefficient is the manufacturer reference data of CAS-Lv). Each automatically segmented liver contour was visually inspected and any inaccurate liver contour was manually corrected by a doctor with over a decade of experience. All patients’ images and liver contours were saved as NII format files.

### Radiomic feature extraction

Before feature extraction, the images are normalized to reduce the voxel spacing variation effect and are resampled with voxel sizes of 1 mm × 1 mm × 1 mm. Parameters are set as follows: normalizeScale: 1000, interpolations: sitkNearestNeighbor, binWidth: 5. For the type of normalization, we adapt the Min-Max normalization method to scale the pixel values of the image. We extracted two radiomics feature sets from CT and MRI, respectively. Each feature set contains 107 features and was split into seven different groups: (1) first-order statistics of voxel intensity features (n = 18), (2) shape features (n = 14), (3) gray level co-occurrence matrix (GLCM) features (n = 24), (4) gray level dependence matrix (GLDM) features (n = 14), (5) gray level run-length matrix(GLRLM) features (n = 16), (6) gray level size zone matrix (GLSZM) features (n = 16), and (7) neighboring gray tone difference matrix (NGTDM) features (n = 5). The feature extraction process is conducted automatically by using the PyRadiomics package (Python version 3.7). Each feature was named by image type, feature group, feature name and concatenated underlines. For example, original_firstorder_Skewness represents the feature ‘Skewness’ extracted from the original image and firstorder group.

### Radiomic feature selection

At first, for each feature, statistical t-test was performed to evaluate differences between different groups. When the two-tailed p-value of the feature was p < 0.1 [19–21], we consider this feature was significantly different between groups and then was retained. Second, to reduce the collinearity of features, spearman correlation analysis was performed. When the correlation coefficient between two features was r>0.9, one feature was randomly retained. At last, the LASSO algorithm was used to reduce the unimportant features and select the features with non-zero coefficient values. The statistical tests, correlation analysis, and LASSO algorithm were implemented by importing the “scipy”, “numpy”, and “sklearn” packages in Python (version 3.7).

### Model construction and performance evaluation

Supervised learning was used for training and prediction. More specifically, five ML algorithms were applied to investigate the performance of the model, whereas these classifiers were Support Vector Machines (SVM), Extra-Trees (ET), Random Forest (RF), Light Gradient Boosting Machine (LightGBM) and eXtreme Gradient Boosting (XGBoost). All selected features were used as input to classify the evaluation of functional liver reserve (ICG-R15 ≤ 10% vs. ICG-R15>10%, ICG-R15 ≤ 20% vs. ICG-R15>20%, and ICG-R15 ≤ 30% vs. ICG-R15>30% as 2-class classifier). All patients were randomly split into two cohorts. One was called the training dataset (70%) and the other was called the test dataset (30%). Each model was trained on the training set and then made predictions by using the test set. A total of five models were constructed and compared with each other to find the best performing model. The ROC curves were used to calculate the AUC value which can evaluate the predictive power of these models. The cut-off values of sensitivity and specificity corresponding to the maximum value of the Youden index were calculated. The final prediction results include AUC (95% CI), ACC, sensitivity and specificity. The AUC value was mainly used to evaluate the performance of classification models. We considered the model with the highest AUC as the best model. The detailed model reconstruction and results calculation were achieved with the aid of “pandas” and “sklearn” packages in Python (version 3.7). The detailed hyper-parameters for ML algorithm are shown in Table 2. Additional details of the models are shown in Supplementary S1.

**Table 2:** The details of hyper-parameters for ML algorithm

Num	Algorithms	Hyper-parameters
1	SVM	kernel=’rbf’, degree = 3, C = 1.0, probability = True
2	Random Forest	n_estimators = 10, max_depth = None, min_samples_split = 2
3	Extra Trees	n_estimators = 10, max_depth = None, min_samples_split = 2
4	XGBoost	n_estimators = 10, max_depth = 5
5	LightGBM	n_estimators = 10, max_depth = 4

The definitions of ACC, sensitivity and specificity are as follows:

$$ACC=\frac{TP+TN}{TP+TN+FP+FN}$$

$$\text{S}\text{e}\text{n}\text{s}\text{i}\text{t}\text{i}\text{v}\text{i}\text{t}\text{y}=\frac{TP}{TP+FN}$$

$$\text{S}\text{p}\text{e}\text{c}\text{i}\text{f}\text{i}\text{c}\text{i}\text{t}\text{y}=\frac{TN}{TN+FP}$$

Where TP/TN is the true positive/negative value, FP/FN is the false positive/negative value.

### Statistical analysis

We used the statistical t-test and Chi-square test to analyze the between-group differences in clinical baseline characteristics (shown in Table 1). Statistical significance was defined as a two-sided p-value < 0.05 (see Supplementary Table S1-S6). Referring to the previous studies [19, 20], we used the statistical t-test to analyze and select the radiomics features with significance to be p-value < 0.1. For features with high repeatability, correlation analysis was performed by Spearman correlation analysis. One of the two features was randomly retained when the correlation coefficient between the two was larger than 0.9. Features were further selected by the LASSO method and were finally used to construct the model. The performance of the classification model was mainly measured by the AUC. Statistical analyses were performed using the “One-key AI” platform (http://www.medai.icu/), which is based on Python (version 3.7).

## Results

The features were selected by conducting statistical tests, spearman correlation analysis, and LASSO. And the final selected features and their corresponding LASSO coefficients derived from Gd-EOB-DTPA-enhanced hepatic MRI and contrast-enhanced CT are shown in Table 3.

**Table 3:** Feature selection results and LASSO coefficient

Data	ICG-R15	Features (Training set)	Coefficient
MRI	ICG-R15 ≤ 10% vs. ICG-R15>10%	original_glszm_ZoneVariance	0.105246
		original_shape_SurfaceVolumeRatio	0.015642
		original_firstorder_Minimum	-0.018612
		original_glrlm_LongRunHighGrayLevelEmphasis	-0.063476
		original_shape_Sphericity	-0.118149
	ICG-R15 ≤ 20% vs. ICG-R15>20%	original_firstorder_Kurtosis	0.111839
		original_shape_SurfaceVolumeRatio	0.083486
		original_glszm_ZoneVariance	0.065786
		original_glszm_SmallAreaLowGrayLevelEmphasis	0.063575
		original_glszm_LargeAreaHighGrayLevelEmphasis	0.05589
		original_glcm_InverseVariance	0.034796
		original_ngtdm_Contrast	0.033712
		original_shape_Sphericity	-0.025289
		original_firstorder_Minimum	-0.100263
	ICG-R15 ≤ 30% vs. ICG-R15>30%	original_firstorder_Kurtosis	0.099461
		original_glszm_ZoneVariance	0.055393
		original_glszm_LargeAreaHighGrayLevelEmphasis	0.029222
		original_glszm_SmallAreaLowGrayLevelEmphasis	0.003266
		original_glszm_SizeZoneNonUniformity	-0.000478
		original_glszm_SmallAreaEmphasis	-0.008009
		original_shape_LeastAxisLength	-0.038956
CT	ICG-R15 ≤ 10% vs. ICG-R15>10%	original_glcm_Correlation	0.061806
		original_glszm_ZoneEntropy	0.030831
		original_firstorder_Energy	-0.096018
		original_glszm_SmallAreaEmphasis	-0.109115
		original_firstorder_RootMeanSquared	-0.126984
	ICG-R15 ≤ 20% vs. ICG-R15>20%*	original_firstorder_TotalEnergy	0.397737
		original_shape_SurfaceVolumeRatio	0.316305
		original_firstorder_Kurtosis	0.086403
		original_ngtdm_Strength	0.074451
		original_shape_Maximum3DDiameter	-0.074731
		original_glcm_ClusterShade	-0.088621
		original_firstorder_Energy	-0.091037
		original_glszm_SmallAreaEmphasis	-0.112469
		original_shape_Maximum2DDiameterColumn	-0.12909
		original_firstorder_RootMeanSquared	-0.354511
	ICG-R15 ≤ 30% vs. ICG-R15>30%	original_shape_SurfaceVolumeRatio	0.048541
		original_glszm_SmallAreaLowGrayLevelEmphasis	0.016216
		original_shape_Maximum2DDiameterSlice	-0.009696
		original_firstorder_Energy	-0.010984
		original_firstorder_90Percentile	-0.021431

* Top 10 features are listed in the table for the comparison between group ICG-R15 ≤ 20% and group ICG-R15>20% based on CT images

Under functional liver reserve thresholds (ICG-R15 = 10%, ICG-R15 = 20% and ICG-R15 = 30%), five ML algorithms were used to construct the model and were trained on the training dataset. The trained models were then used to predict the result on the test dataset. The detailed performance of the five models is described in Table 4. For MRI groups, more specifically, the classifier XGBoost achieves the highest performance when ICG-R15 = 10% is used as a threshold, with AUC = 0.917 (95% CI: 0.823–1.000) and ACC = 0.882. And the classifier Random Forest achieves the highest performance with AUC = 0.979 (95% CI: 0.941–1.000) and ACC = 0.882 at threshold ICG-R15 = 20%. For threshold ICG-R15 = 30%, the classifier XGBoost performs the best with AUC = 0.961 (95% CI: 0.890–1.000) and ACC = 0.941. Similar to the results for MRI groups, the classifier XGBoost for CT groups also achieves the best performance when threshold ICG-R15 = 10% (AUC = 0.822, 95% CI: 0.700–0.944, ACC = 0.842), and ICG-R15 = 30% (AUC = 0.938, 95% CI: 0.824–1.000, ACC = 0.965). Under threshold ICG-R15 = 20%, the classifier SVM is observed to perform the best with an AUC value of 0.860 (95% CI: 0.758–0.963) and ACC of 0.842. The detailed information of the best models is listed in Table 5.

**Table 4:** Performance comparison among machine learning models

Data	ICG-R15		SVM	RF	ExtraTrees	XGBoost	LightGBM
MRI	ICG-R15 ≤ 10% vs. ICG-R15>10%	Test set ACC	0.824	0.765	0.824	**0.853**	0.794
		Test set AUC (95%CI)	0.802(0.639–0.965)	0.839(0.703–0.974)	0.873(0.743–1.000)	**0.899(0.784–1.000)**	0.806(0.650–0.962)
	ICG-R15 ≤ 20% vs. ICG-R15>20%	Test set ACC	0.824	**0.882**	0.735	0.824	0.824
		Test set AUC (95%CI)	0.893(0.780–1.000)	**0.979(0.941–1.000)**	0.878(0.739–1.000)	0.946(0.866–1.000)	0.833(0.632–1.000)
	ICG-R15 ≤ 30% vs. ICG-R15>30%	Test set ACC	0.882	0.618	0.882	**0.941**	0.794
		Test set AUC (95%CI)	0.922(0.802–1.000)	0.789(0.481–1.000)	0.945(0.866–1.000)	**0.961(0.890–1.000)**	0.891(0.743–1.000)
CT	ICG-R15 ≤ 10% vs. ICG-R15>10%	Test set ACC	0.772	0.632	0.667	**0.842**	0.702
		Test set AUC (95%CI)	0.734(0.590–0.879)	0.661(0.514–0.807)	0.723(0.576–0.870)	**0.822(0.700–0.944)**	0.741(0.610–0.872)
	ICG-R15 ≤ 20% vs. ICG-R15>20%	Test set ACC	**0.842**	0.667	0.702	0.684	0.684
		Test set AUC (95%CI)	**0.860(0.758–0.963)**	0.722(0.591–0.853)	0.634(0.478–0.789)	0.709(0.570–0.847)	0.692(0.552–0.832)
	ICG-R15 ≤ 30% vs. ICG-R15>30%	Test set ACC	0.982	0.912	0.807	**0.965**	0.982
		Test set AUC (95%CI)	0.865(0.600–1.000)	0.871(0.683–1.000)	0.783(0.471–1.000)	**0.938(0.824–1.000)**	0.925(0.776–1.000)

The performance of the best model is in boldface

**Table 5:** Performance of the best MRI- and CT-based machine learning classification model

Data	ICG-R15	Cohort	AUC (95%CI)	Accuracy	Sensitivity	Specificity	model
MRI	ICG-R15 ≤ 10% vs. ICG-R15>10%	Training	0.996(0.989–1.000)	0.987	0.980	1.000	XGBoost
		Test	0.899(0.784–1.000)	0.853	0.875	0.833	XGBoost
	ICG-R15 ≤ 20% vs. ICG-R15>20%	Training	0.995(0.986–1.000)	0.962	0.929	0.980	Random Forest
		Test	0.979(0.941–1.000)	0.882	1.000	0.857	Random Forest
	ICG-R15 ≤ 30% vs. ICG-R15>30%	Training	0.997(0.991–1.000)	0.962	1.000	0.951	XGBoost
		Test	0.961(0.890–1.000)	0.941	1.000	0.968	XGBoost
CT	ICG-R15 ≤ 10% vs. ICG-R15>10%	Training	0.998(0.995–1.000)	0.970	0.957	1.000	XGBoost
		Test	0.822(0.700–0.944)	0.842	0.917	0.714	XGBoost
	ICG-R15 ≤ 20% vs. ICG-R15>20%	Training	0.866(0.781–0.951)	0.842	0.872	0.830	SVM
		Test	0.860(0.758–0.963)	0.842	0.840	0.844	SVM
	ICG-R15 ≤ 30% vs. ICG-R15>30%	Training	0.997(0.991–1.000)	0.992	1.000	0.991	XGBoost
		Test	0.938(0.824–1.000)	0.965	0.800	0.981	XGBoost

ICG-R15: indocyanine green retention rate at 15 min, AUC: Area under the ROI curve, ACC: Accuracy

**Fig. 2:**
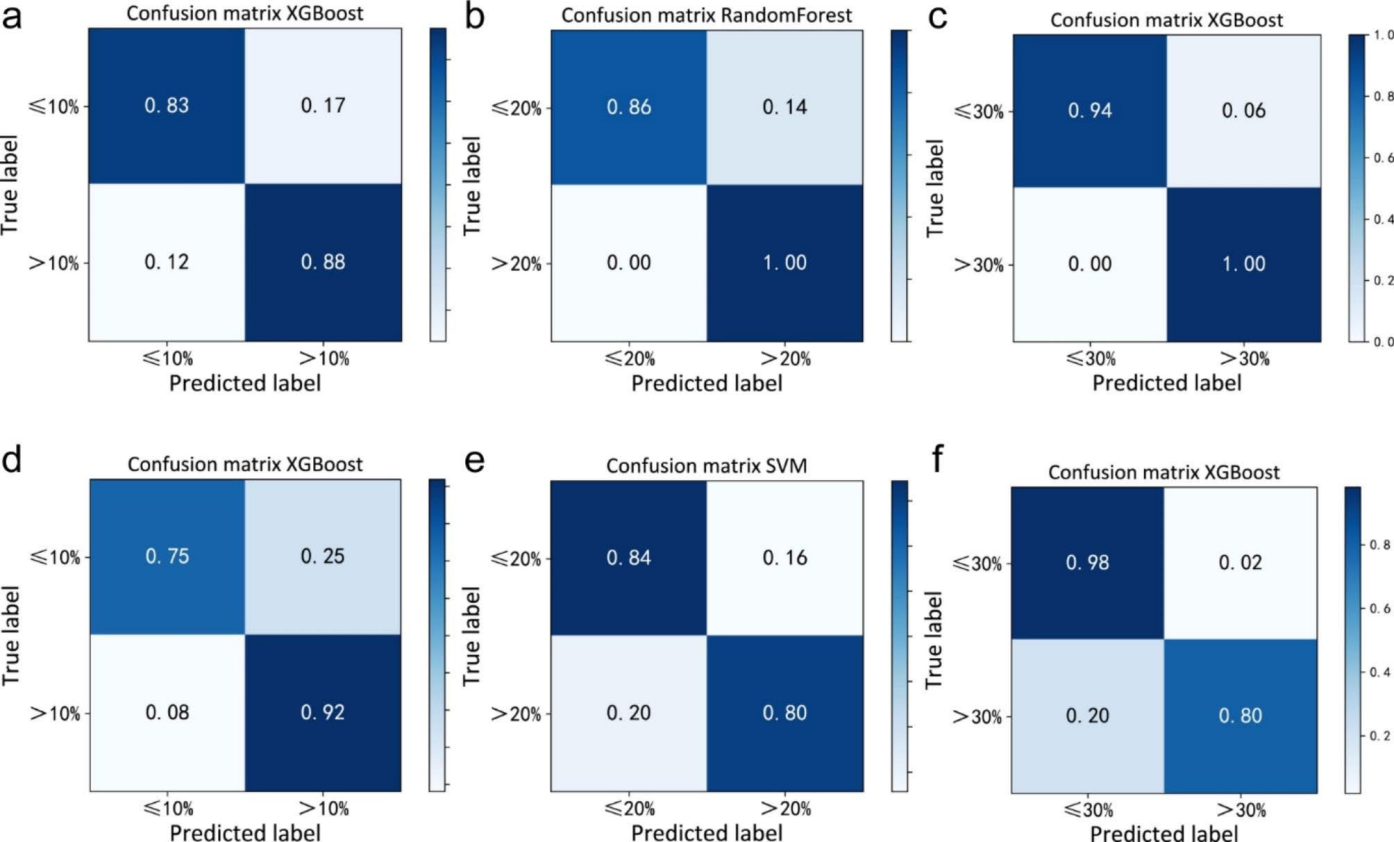
Based upon Gd-EOB-DTPA-enhanced hepatic MRI, **a** XGBoost Confusion matrix when ICG-R15 = 10% was selected as a threshold; **b** Random Forest Confusion matrix when ICG-R15 = 20% was selected as a threshold; **c** XGBoost Confusion matrix when ICG-R15 = 30% was selected as a threshold. Based upon contrast-enhanced CT, **d** XGBoost Confusion matrix when ICG-R15 = 10% was selected as a threshold; **e** SVM Confusion matrix when ICG-R15 = 20% was selected as a threshold; **f** XGBoost Confusion matrix when ICG-R15 = 30% was selected as a threshold

**Fig. 3:**
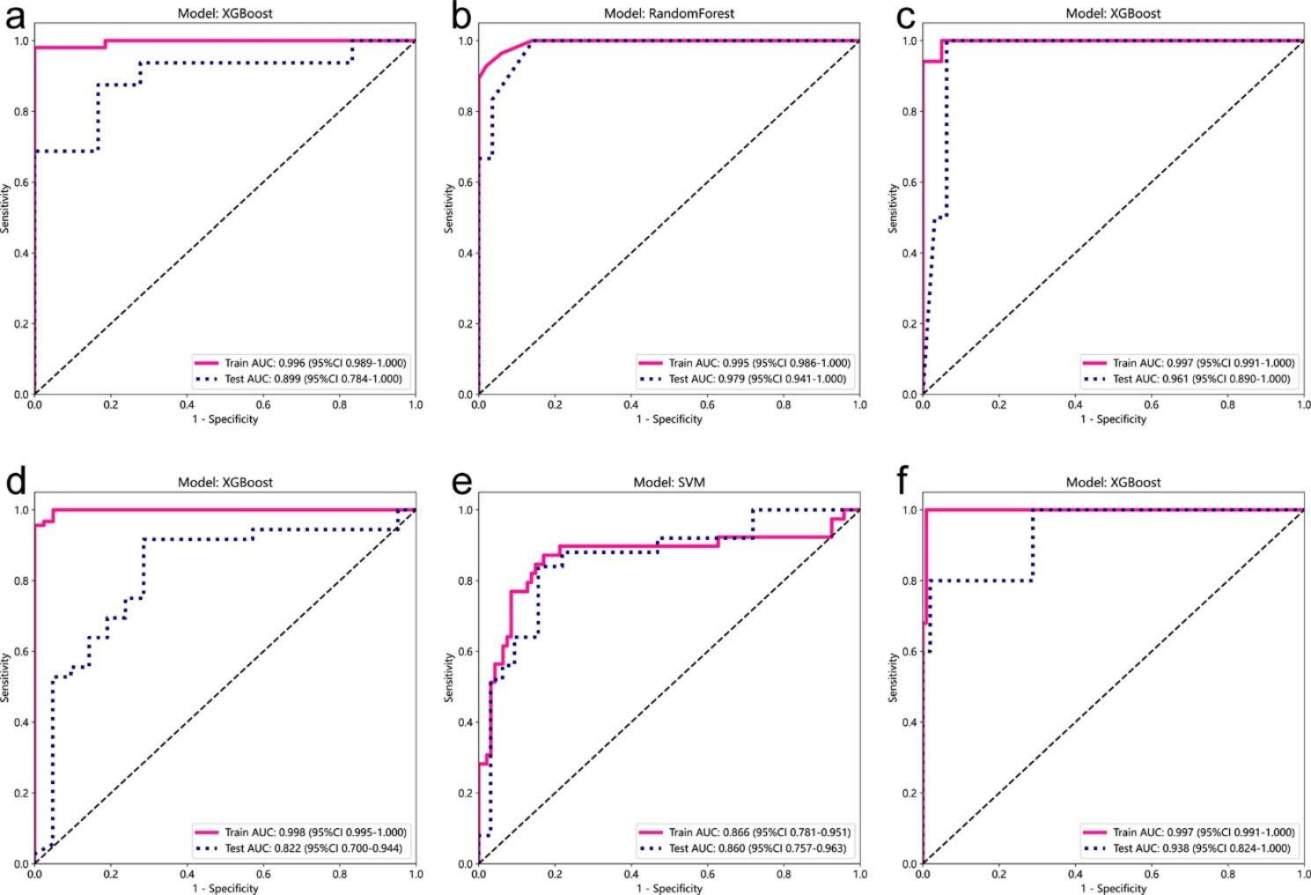
Based upon Gd-EOB-DTPA-enhanced hepatic MRI, **a** XGBoost ROC curve when ICG-R15 = 10% was selected as a threshold; **b** Random Forest ROC curve when ICG-R15 = 20% was selected as a threshold; **c** XGBoost ROC curve when ICG-R15 = 30% was selected as a threshold. Based upon contrast-enhanced CT, **d** XGBoost ROC curve when ICG-R15 = 10% was selected as a threshold; **e** SVM ROC curve when ICG-R15 = 20% was selected as a threshold; **f** XGBoost ROC curve when ICG-R15 = 30% was selected as a threshold

The model confusion matrices and ROC curves are shown in Figs. 2 and 3, respectively. All AUC values are greater than 0.89 for the test dataset from MRI and are greater than 0.82 for the test dataset from CT. The results indicate that both MRI-based and CT-based ML models can achieve the goal of classification in distinguishing the different values of ICG-R15 to some extent, which is promising to become an additional method to predict the functional liver reserve.

## Discussion

Radiomics has shown great value in the diagnosis and therapy of multiple diseases. We consider whether it is possible to use the radiomics method to perform an accurate assessment of functional liver reserve based on Gd-EOB-DTPA-enhanced hepatic MRI and contrast-enhanced CT in HCC patients. Under this perspective, MRI-based and CT-based ML models are developed and validated for distinguishing patients with functional liver reserves of different states. Our results demonstrate that both MRI-based and CT-based models worked satisfactorily in the aspect of the assessment of functional liver reserve.

Recently, there has been an increasing application of imaging techniques in measuring the hepatic function in HCC patients, because it can provide more significant clinical information than an overall assessment [22]. For example, previous studies based on medical image analysis have demonstrated that liver has regional differences in hepatic parenchymal abnormalities [23, 24]. One recent work from Zhaoqi Shi et al. shows that radiomics analysis can be applied in the preoperative assessment of functional liver reserve in HCC patients [25]. However, this research only focused on Gd-EOB-DTPA-enhanced hepatic MRI to predict the ICG classification value to evaluate liver function in HCC patients, and the functional liver reserve thresholds are set to be ICG-R15 = 10%, ICG-R15 = 15%, and ICG-R15 = 20%. The analysis of the CT images is not mentioned. In our work, considering that the contrast-enhanced CT is regarded as the most common modality for patients with HCC, we add CT images to assess functional liver reserve. And we set functional liver reserve thresholds to be ICG-R15 = 10%, ICG-R15 = 20%, and ICG-R15 = 30% according to the requirement of clinical surgical strategies. By referring to the criteria of safe hepatic resection proposed by Makuuchi [9], if ICG-R15<10%, trisegmentectomy and bisegmentectomy of the liver can be performed; If 10%≤ICG-R15<20%, left lobictomy right monosegmentectomy of the liver can be performed; If 20%≤ICG-R15<30%, subsegmentectomy of Couinaud of the liver can be performed; If ICG-R15 ≥ 30%, it is necessary to limit resection or enucleation liver for transplantation [9]. Compared with Zhaoqi’s work, our thresholds are larger in general and are in accordance with the mentioned Makuuchi criteria for safe hepatic resection, which can provide a better reference for clinical surgery. By developing the radiomics model, we could assist doctors to evaluate functional liver reserve in the hospital without ICG equipment.

In our work, Gd-EOB-DTPA-enhanced hepatic MRI and contrast-enhanced CT are selected as data for evaluating presurgical liver function. The hepatobiliary phase after 15 min from Gd-EOB-DTPA-enhanced hepatic MRI is further selected. During hepatobiliary phase, HCCs appear hypointense in Gd-EOB-DTPA-enhanced images because of contrast medium discharges into hepatocytes and bile ducts. Hence, the diagnostic sensitivity and specificity of HCC are dramatically improved [26]. The obtained signals during Gd-EOB-DTPA-enhanced MRI imaging can describe anatomical characteristics of liver and hepatocyte-specific function [26]. And the effectiveness of Gd-EOB-DTPA-enhanced hepatic MRI for evaluating liver function has been evaluated in several works [27]. Besides, for contrast-enhanced CT, we select the portal venous phase to evaluate the presurgical liver function. Intense contrast uptake in the arterial phase was conducted before extracellular contrast washout in portal venous and/or delayed phases [28]. During the portal venous phase, owing to the washout, the CT value of HCCs was lower than liver parenchyma which is beneficial to observe the radiomics features about liver function. Washout here means a relative decrease in the extracellular contrast to the background of the portal venous phase and/or delayed phases [29–32].

In our research, the features in different threshold groups were selected based on shape, voxel intensity, and texture to predict classification. The developed disaggregated model was too complex for clinical practice since it comprises many features. Further research is required to simplify the features and thus we used five classifiers for modeling. Among all classifiers, XGBoost was demonstrated to perform best. The XGBoost is an improved model based on the gradient boosted decision tree (GBDT). It is an ensemble learning method that combines the predictions of multiple weak models to produce a stronger prediction [33]. The XGBoost uses both LASSO and Ridge Regression regularization to penalize the highly complex model and also uses built-in cross-validation to help the algorithm prevent overfitting. The XGBoost has become one of the widely used ML algorithms due to its state-of-the-art performance in many ML tasks, such as classification and regression [34].

However, several limitations should be mentioned in our study. Firstly, the provided research images were obtained from our center, more patients are needed in order to achieve external cohort validation. The baseline characteristics and features from a single center may not conform to the population. Secondly, the analysis of functional liver reserve under specific thresholds is a 2-class classification problem, which cannot cover all of the clinically significant ICG-R15 value intervals mentioned in the Makuuchi criteria for safe hepatic resection [9]. Thirdly, regarding the fact that some patients had CT scans but no MRI scans, we did not make the multimodality evaluation with CT and MRI. In the future, the classification with massive patients and multi-center data will be investigated by different ICG value intervals and multi-class classification methods. The multimodality evaluation with CT, MRI and clinical data will be a focus for our follow-up studies.

## Conclusion

Both MRI-based and CT-based ML models are shown to achieve the goal of classification in distinguishing the different values of ICG-R15 to some extent, which are proved to be valuable methods for functional liver reserve evaluation. Among the five classifiers, XGBoost was demonstrated to perform best. Our work provides valuable insights, which can help clinicians to construct an effective prediction model and develop personalized precision treatment strategies.

## Electronic supplementary material

Below is the link to the electronic supplementary material.

Supplementary Material 1

## Data Availability

The data and materials in this study are available through the corresponding authors with permission of the Affiliated Hospital of Qingdao University.

## Abbreviations

ACC: Accuracy
AST: Aspartate aminotransferase
ALT: Alanine transaminase
PT: Prothrombin time
CT: Computed tomography
CI: Confidence intervals
MRI: Magnetic resonance imaging
HCC: Hepatocellular carcinoma
RFA: Radiofrequency ablation
ET: Extra-Trees
Gd-EOB-DTPA: Gadolinium ethoxybenzyl dimeglumine
ML: Machine learning
ICG-R15: Indocyanine green retention rate at 15 min
ROI: Regions of interest
RF: Random Forest
AUC: Area under the ROI curve
ROC: Receiver operating characteristic
BMI: Body Mass Index
HBV: Hepatitis B Virus
ALB: Albumin
TBIL: Total Bilirubin
GGT: Gamma-glutamyltransferase
SVM: Support Vector Machines
LASSO: Least absolute shrinkage and selection operator
XGBoost: eXtreme Gradient Boosting
LightGBM: Light Gradient Boosting Machine
