# Supplementary material for: Machine learning-based radiomics analysis of preoperative functional liver reserve with MRI and CT image
Source: BMC Med Imaging. 2023 Jul 17;23:94. doi: 10.1186/s12880-023-01050-1 (PMC10353100; doi:10.1186/s12880-023-01050-1)
Supplement: Supplementary file 1 — Supplementary Material 1 [file 12880_2023_1050_MOESM1_ESM.docx]

**Supplementary S1**

The Support vector machine (SVM) is a set of supervised learning methods used for [classification](https://scikit-learn.org/stable/modules/svm.html#svm-classification), [regression](https://scikit-learn.org/stable/modules/svm.html#svm-regression) and [outliers detection](https://scikit-learn.org/stable/modules/svm.html#svm-outlier-detection). Its boundary decision is to solve the maximum-margin hyperplane for the learned samples [1]. The loss function used in the SVM is the hinge loss function, SVM uses the hinge loss function to calculate empirical risk and adds regularization term to the solving system to optimize structural risk. SVM is a classifier with sparsity and robustness. SVM, one of the common kernel learning methods, can be used for nonlinear classification by kernel method [2].

The Random Forest (RF) is a bagging-based machine learning model that applies an ensemble learning technique by constructing a group of decision trees [3]. Compared with OLS that assumes a linear relation, RF can model nonlinear relations between input features and the target variable. Given this ability, RF has been used in a variety of previous studies in which the input features and the target variable likely have a nonlinear relation [4,5].

The ExtraTrees is a variant of the Random Forest. The Extra Tree algorithm is an ensemble of randomized decision trees on various sub-samples of the dataset [6,7]. The Generalizing capability of ExtraTrees is better than Random Forest.

The XGBoost is an improved model based on the gradient boosted decision tree (GBDT). It is an ensemble learning method that combines the predictions of multiple weak models to produce a stronger prediction [8]. The XGBoost uses both LASSO and Ridge Regression regularization to penalize the highly complex model, and XGBoost also uses built-in cross-validation to help the algorithm prevents overfitting. The XGBoost has become one of the widely used machine learning algorithms due to its state-of-the-art performance in many machine learning tasks, such as classification and regression [9].

The LightGBM is a gradient boosting framework that uses tree based learning algorithms. It is designed to be distributed and efficient with the following advantages: Faster training speed and higher efficiency; Lower memory usage; Better accuracy; Support of parallel, distributed, and GPU learning; Capable of handling large-scale data [10].

The details of hyper-parameters for ML algorithm that we used in our work are shown in Table 2 in the main text.

**Reference**

1. Vapnik, V.．Statistical learning theory. 1998 (Vol. 3). New York, NY: Wiley，1998：Chapter 10-11, pp.401-492

2. Xi Y, Liu J, Shen G. Low expression of IGFBP4 and TAGLN accelerate the poor overall survival of osteosarcoma. Sci Rep. 2022;12(1):9298. Published 2022 Jun 3.

3. Liaw A, Wiener M. Classifcation and regression by randomForest. R News. 2002; 2:18–22.

4. Huang X, Lu J, Gao S, Wang S, Liu Z, Wei H. Staying at home is a privilege: evidence from fne-grained mobile phone location data in the United States during the COVID-19 pandemic. Ann Am Assoc Geogr. 2021.

5. Xia Z, Stewart K, Fan J. Incorporating space and time into random forest models for analyzing geospatial patterns of drug-related crime incidents in a major us metropolitan area. Comput Environ Urban Syst. 2021; 87:101599.

6. Pedregosa F, Varoquaux G, Gramfort A, Michel V, Thirion B, Grisel O, Blondel M, Prettenhofer P, Weiss R, Dubourg V. Scikit-learn: Machine learning in Python. J Mach Learn Res. 2011; 12:2825–30.

7. van Buuren S, Groothuis-Oudshoorn K. mice: multivariate imputation by chained equations in R. J Stat Soft. 2010; 45:1–68.

8. Chen T, Guestrin C. XGBoost: A Scalable Tree Boosting System. ACM, 2016.

9. Wang C, Guo J. A data-driven framework for learners' cognitive load detection using ECG-PPG physiological feature fusion and XGBoost classification. Procedia Computer Science, 2019, 147:338-348.

10. Qi M. LightGBM: A Highly Efficient Gradient Boosting Decision Tree. Neural Information Processing Systems. Curran Associates Inc. 2017.

**Supplementary Table S1** Baseline clinical characteristics of patients in the cohort (CT: ICG-R15≤10% vs ICG-R15＞10%).

| **Characteristics (CT)** | **Training cohort (n=133)** | | **p-value** | **Test cohort (n=57)** | | **p-value** |
| --- | --- | --- | --- | --- | --- | --- |
|  | **train-ICG-R15≤10%(42)** | **train-ICG-R15＞10%(91)** |  | **test-ICG-R15≤10%(20)** | **test-ICG-R15＞10%(37)** |  |
| Age (mean ± SD) y | 56.93±9.03 | 59.92±8.90 | 0.077 | 58.62±7.72 | 61.44±9.23 | 0.243 |
| BMI (mean ± SE) kg/m^2^ | 25.03±3.70 | 24.81±4.13 | 0.768 | 24.54±2.51 | 24.39±3.70 | 0.869 |
| ALT (mean ± SE) IU/L | 33.69±27.95 | 55.28±78.91 | 0.091 | 35.43±37.36 | 32.74±26.66 | 0.753 |
| AST (mean ± SE) IU/L | 33.92±29.77 | 58.88±63.74 | 0.018 | 38.88±65.17 | 47.31±44.28 | 0.564 |
| GGT (mean ± SE) IU/L | 56.67±49.49 | 122.01±170.70 | 0.018 | 69.82±75.58 | 93.88±87.25 | 0.297 |
| TBIL (mean ± SE) µmol/L | 14.97±7.73 | 24.01±13.56 | <0.001 | 16.03±6.75 | 20.21±8.95 | 0.070 |
| ALB (mean ± SE) g/L | 41.15±3.23 | 37.17±4.96 | <0.001 | 40.42±3.58 | 35.82±4.67 | <0.001 |
| PT (mean ± SE) s | 11.00±1.35 | 11.77±1.56 | 0.007 | 10.86±1.26 | 11.51±1.31 | 0.073 |
| Gender (%) |  |  | 0.394 |  |  | 0.022 |
| Female | 8(19.51) | 26(28.26) |  | null | 10(27.78) |  |
| Male | 33(80.49) | 66(71.74) |  | 21(100.00) | 26(72.22) |  |
| HBV infection (%) |  |  | 0.197 |  |  | 0.076 |
| No | 1(2.44) | 10(10.87) |  | 1(4.76) | 10(27.78) |  |
| Yes | 40(97.56) | 82(89.13) |  | 20(95.24) | 26(72.22) |  |
| Liver cirrhosis (%) |  |  | 0.087 |  |  | 0.382 |
| No | 20(48.78) | 29(31.52) |  | 5(23.81) | 14(38.89) |  |
| Yes | 21(51.22) | 63(68.48) |  | 16(76.19) | 22(61.11) |  |

**Supplementary Table S2** Baseline clinical characteristics of patients in the cohort (CT: ICG-R15≤20% vs ICG-R15＞20%).

| **Characteristics (CT)** | **Training cohort (n=133)** | | **p-value** | **Test cohort (n=57)** | | **p-value** |
| --- | --- | --- | --- | --- | --- | --- |
|  | **train-ICG-R15≤20%(94)** | **train-ICG-R15＞20%(39)** |  | **test-ICG-R15≤20%(32)** | **test-ICG-R15＞20%(25)** |  |
| Age (mean ± SD) y | 60.30±9.61 | 57.72±8.04 | 0.143 | 58.00±8.17 | 60.60±8.36 | 0.243 |
| BMI (mean ± SE) kg/m^2^ | 24.69±3.50 | 25.10±3.59 | 0.540 | 24.30±3.24 | 24.98±5.56 | 0.566 |
| ALT (mean ± SE) IU/L | 38.18±39.75 | 58.29±100.16 | 0.099 | 36.28±33.54 | 54.65±62.56 | 0.161 |
| AST (mean ± SE) IU/L | 42.68±49.81 | 64.14±70.96 | 0.049 | 34.82±20.99 | 67.98±69.35 | 0.013 |
| GGT (mean ± SE) IU/L | 93.17±136.81 | 102.06±142.25 | 0.737 | 96.49±121.68 | 102.74±111.50 | 0.843 |
| TBIL (mean ± SE) µmol/L | 17.23±7.97 | 27.69±14.98 | <0.001 | 16.47±7.96 | 26.41±14.21 | 0.001 |
| ALB (mean ± SE) g/L | 39.25±4.34 | 35.58±5.19 | <0.001 | 40.58±3.75 | 34.77±4.00 | <0.001 |
| PT (mean ± SE) s | 11.17±1.16 | 12.28±1.73 | <0.001 | 10.75±1.34 | 12.15±1.52 | <0.001 |
| Gender (%) |  |  | 0.218 |  |  | 0.399 |
| Female | 18(19.15) | 12(30.77) |  | 6(18.75) | 8(32.00) |  |
| Male | 76(80.85) | 27(69.23) |  | 26(81.25) | 17(68.00) |  |
| HBV infection (%) |  |  | 0.387 |  |  | 0.772 |
| No | 10(10.64) | 7(17.95) |  | 2(6.25) | 3(12.00) |  |
| Yes | 84(89.36) | 32(82.05) |  | 30(93.75) | 22(88.00) |  |
| Liver cirrhosis (%) |  |  | 0.116 |  |  | 0.013 |
| No | 42(44.68) | 11(28.21) |  | 13(40.62) | 2(8.00) |  |
| Yes | 52(55.32) | 28(71.79) |  | 19(59.38) | 23(92.00) |  |

**Supplementary Table S3** Baseline clinical characteristics of patients in the cohort (CT: ICG-R15≤30% vs ICG-R15＞30%).

| **Characteristics (CT)** | **Training cohort (n=133)** | | **p-value** | **Test cohort (n=57)** | | **p-value** |
| --- | --- | --- | --- | --- | --- | --- |
|  | **train-ICG-R15≤30%(108)** | **train-ICG-R15＞30%(25)** |  | **test-ICG-R15≤30%(52)** | **test-ICG-R15＞30%(5)** |  |
| Age (mean ± SD) y | 59.91±9.18 | 59.56±7.74 | 0.861 | 59.17±8.87 | 50.80±7.29 | 0.046 |
| BMI (mean ± SE) kg/m^2^ | 24.65±3.45 | 24.44±3.16 | 0.786 | 24.41±3.25 | 31.90±10.01 | <0.001 |
| ALT (mean ± SE) IU/L | 40.84±43.13 | 65.48±117.76 | 0.084 | 42.21±49.76 | 29.40±15.18 | 0.572 |
| AST (mean ± SE) IU/L | 47.96±56.37 | 66.52±68.47 | 0.157 | 44.13±47.86 | 38.00±8.77 | 0.778 |
| GGT (mean ± SE) IU/L | 96.38±117.13 | 83.31±88.16 | 0.601 | 95.92±146.42 | 183.00±346.69 | 0.276 |
| TBIL (mean ± SE) µmol/L | 18.24±8.34 | 30.01±15.19 | <0.001 | 18.75±12.40 | 38.32±8.27 | 0.001 |
| ALB (mean ± SE) g/L | 39.04±4.60 | 33.91±4.52 | <0.001 | 38.74±4.37 | 33.33±3.22 | 0.010 |
| PT (mean ± SE) s | 11.25±1.23 | 12.64±1.80 | <0.001 | 11.07±1.33 | 13.82±1.29 | <0.001 |
| Gender (%) |  |  | 0.101 |  |  | 0.845 |
| Female | 20(18.52) | 9(36.00) |  | 13(25.00) | 2(40.00) |  |
| Male | 88(81.48) | 16(64.00) |  | 39(75.00) | 3(60.00) |  |
| HBV infection (%) |  |  | 0.043 |  |  | 0.785 |
| No | 11(10.19) | 7(28.00) |  | 3(5.77) | 1(20.00) |  |
| Yes | 97(89.81) | 18(72.00) |  | 49(94.23) | 4(80.00) |  |
| Liver cirrhosis (%) |  |  | 0.074 |  |  | 0.937 |
| No | 45(41.67) | 5(20.00) |  | 17(32.69) | 1(20.00) |  |
| Yes | 63(58.33) | 20(80.00) |  | 35(67.31) | 4(80.00) |  |

**Supplementary Table S4** Baseline clinical characteristics of patients in the cohort (MR: ICG-R15≤10% vs ICG-R15＞10%).

| **Characteristics (MR)** | **Training cohort (n=78)** | | **p-value** | **Test cohort (n=34)** | | **p-value** |
| --- | --- | --- | --- | --- | --- | --- |
|  | **train-ICG-R15≤10%(33)** | **train-ICG-R15＞10%(45)** |  | **test-ICG-R15≤10%(12)** | **test-ICG-R15＞10%(22)** |  |
| Age (mean ± SD) y | 56.70±8.10 | 59.76±7.99 | 0.101 | 55.92±9.77 | 60.64±9.58 | 0.182 |
| BMI (mean ± SE) kg/m^2^ | 25.50±3.05 | 24.64±3.45 | 0.258 | 23.42±3.24 | 25.96±6.52 | 0.216 |
| ALT (mean ± SE) IU/L | 40.73±39.97 | 45.05±82.61 | 0.782 | 35.00±23.02 | 35.95±30.02 | 0.924 |
| AST (mean ± SE) IU/L | 47.72±69.60 | 43.29±37.82 | 0.719 | 30.67±10.47 | 41.73±28.22 | 0.202 |
| GGT (mean ± SE) IU/L | 76.08±89.15 | 92.60±116.79 | 0.499 | 54.83±67.63 | 104.77±154.98 | 0.298 |
| TBIL (mean ± SE) µmol/L | 16.29±8.71 | 19.81±10.98 | 0.132 | 13.22±4.48 | 22.82±11.65 | 0.010 |
| ALB (mean ± SE) g/L | 39.40±4.08 | 37.43±5.16 | 0.074 | 39.59±2.79 | 38.53±5.44 | 0.533 |
| PT (mean ± SE) s | 10.97±1.33 | 11.61±1.61 | 0.069 | 10.71±1.08 | 11.68±1.47 | 0.053 |
| Gender (%) |  |  | 0.751 |  |  | 0.922 |
| Female | 10(30.30) | 11(24.44) |  | 1(8.33) | 3(13.64) |  |
| Male | 23(69.70) | 34(75.56) |  | 11(91.67) | 19(86.36) |  |
| HBV infection (%) |  |  | 0.719 |  |  | 0.068 |
| No | 3(9.09) | 2(4.44) |  | 3(25.00) | null |  |
| Yes | 30(90.91) | 43(95.56) |  | 9(75.00) | 22(100.00) |  |
| Liver cirrhosis (%) |  |  | 0.745 |  |  | 0.501 |
| No | 9(27.27) | 15(33.33) |  | 6(50.00) | 7(31.82) |  |
| Yes | 24(72.73) | 30(66.67) |  | 6(50.00) | 15(68.18) |  |

**Supplementary Table S5** Baseline clinical characteristics of patients in the cohort (MR: ICG-R15≤20% vs ICG-R15＞20%).

| **Characteristics (MR)** | **Training cohort (n=78)** | | **p-value** | **Test cohort (n=34)** | | **p-value** |
| --- | --- | --- | --- | --- | --- | --- |
|  | **train-ICG-R15≤20%(50)** | **train-ICG-R15＞20%(28)** |  | **test-ICG-R15≤20%(28)** | **test-ICG-R15＞20%(6)** |  |
| Age (mean ± SD) y | 57.02±9.11 | 61.04±8.29 | 0.074 | 60.42±8.61 | 56.82±5.10 | 0.205 |
| BMI (mean ± SE) kg/m^2^ | 24.70±3.23 | 25.94±6.33 | 0.267 | 24.58±2.85 | 25.66±4.92 | 0.402 |
| ALT (mean ± SE) IU/L | 36.01±34.41 | 54.78±112.00 | 0.273 | 32.23±16.81 | 55.64±49.48 | 0.037 |
| AST (mean ± SE) IU/L | 42.46±57.25 | 45.04±39.46 | 0.845 | 34.26±19.39 | 61.27±46.79 | 0.017 |
| GGT (mean ± SE) IU/L | 63.45±69.16 | 115.64±168.51 | 0.060 | 79.88±88.05 | 145.91±168.61 | 0.125 |
| TBIL (mean ± SE) µmol/L | 15.87±6.42 | 22.38±12.58 | 0.004 | 19.17±12.25 | 22.83±12.41 | 0.413 |
| ALB (mean ± SE) g/L | 39.42±3.72 | 35.51±5.83 | <0.001 | 40.06±4.75 | 36.31±3.47 | 0.024 |
| PT (mean ± SE) s | 11.11±1.36 | 11.73±1.96 | 0.118 | 11.20±1.26 | 11.93±1.23 | 0.114 |
| Gender (%) |  |  | 0.330 |  |  | 0.883 |
| Female | 9(17.31) | 7(30.43) |  | 6(23.08) | 3(27.27) |  |
| Male | 43(82.69) | 16(69.57) |  | 20(76.92) | 8(72.73) |  |
| HBV infection (%) |  |  | 0.216 |  |  | 0.880 |
| No | 6(11.54) | null |  | 2(7.69) | null |  |
| Yes | 46(88.46) | 23(100.00) |  | 24(92.31) | 11(100.00) |  |
| Liver cirrhosis (%) |  |  | 0.803 |  |  | 0.544 |
| No | 18(34.62) | 8(34.78) |  | 9(34.62) | 2(18.18) |  |
| Yes | 34(65.38) | 15(65.22) |  | 17(65.38) | 9(81.82) |  |

**Supplementary Table S6** Baseline clinical characteristics of patients in the cohort (MR: ICG-R15≤30% vs ICG-R15＞30%).

| **Characteristics (MR)** | **Training cohort (n=78)** | | **p-value** | **Test cohort (n=34)** | | **p-value** |
| --- | --- | --- | --- | --- | --- | --- |
|  | **train-ICG-R15≤30%(59)** | **train-ICG-R15＞30%(17)** |  | **test-ICG-R15≤30%(34)** | **test-ICG-R15＞30%(2)** |  |
| Age (mean ± SD) y | 58.80±8.38 | 59.29±8.22 | 0.831 | 57.84±9.70 | 59.50±2.12 | 0.813 |
| BMI (mean ± SE) kg/m^2^ | 25.18±3.45 | 24.45±3.96 | 0.457 | 24.42±3.71 | 34.69±17.25 | 0.006 |
| ALT (mean ± SE) IU/L | 41.55±72.47 | 45.88±34.62 | 0.812 | 34.56±31.96 | 81.00±83.44 | 0.076 |
| AST (mean ± SE) IU/L | 41.32±47.45 | 49.76±33.69 | 0.495 | 39.25±48.67 | 93.00±73.54 | 0.147 |
| GGT (mean ± SE) IU/L | 71.31±83.84 | 155.88±207.11 | 0.013 | 79.43±84.99 | 49.50±40.31 | 0.628 |
| TBIL (mean ± SE) µmol/L | 18.34±10.30 | 20.27±12.55 | 0.518 | 17.43±7.41 | 34.22±24.88 | 0.011 |
| ALB (mean ± SE) g/L | 39.55±4.05 | 35.61±5.03 | 0.001 | 38.22±5.15 | 33.39±5.24 | 0.207 |
| PT (mean ± SE) s | 11.31±1.45 | 11.62±1.14 | 0.410 | 11.12±1.52 | 13.40±3.68 | 0.064 |
| Gender (%) |  |  | 0.041 |  |  | 0.961 |
| Female | 9(14.75) | 7(41.18) |  | 8(25.00) | 1(50.00) |  |
| Male | 52(85.25) | 10(58.82) |  | 24(75.00) | 1(50.00) |  |
| HBV infection (%) |  |  | 0.826 |  |  | 0.672 |
| No | 3(4.92) | null |  | 5(15.62) | null |  |
| Yes | 58(95.08) | 17(100.00) |  | 27(84.38) | 2(100.00) |  |
| Liver cirrhosis (%) |  |  | 0.299 |  |  | 0.575 |
| No | 15(24.59) | 7(41.18) |  | 15(46.88) | null |  |
| Yes | 46(75.41) | 10(58.82) |  | 17(53.12) | 2(100.00) |  |

ALT: alanine transaminase, BMI: Body Mass Index, HBV: Hepatitis B Virus; TBIL: total Bilirubin, ALB: albumin, PT: prothrombin time, AST: aspartate aminotransferase, GGT: gamma-glutamyltransferase, ICG-R15: indocyanine green retention rate at 15 min, y: years.
